# Adult quality of life patterns and trajectories during the COVID-19 pandemic in Germany

**DOI:** 10.1007/s12144-022-03628-4

**Published:** 2022-09-30

**Authors:** Caroline Cohrdes, Britta Wetzel, Rüdiger Pryss, Harald Baumeister, Kristin Göbel

**Affiliations:** 1 Mental Health Research Unit, Department of Epidemiology and Health Monitoring, Robert Koch Institute Berlin, PO Box 650261, D-13302 Berlin, Germany; 2 Institute of Clinical Epidemiology and Biometry, University of Würzburg, Würzburg, Germany; 3 Department of Clinical Psychology and Psychotherapy, Ulm University, Ulm, Germany; 4 Division of Developmental Psychology, Department of Education and Psychology, Freie Universität Berlin, Berlin, Germany

**Keywords:** Quality of life, COVID-19, Latent class analysis, Longitudinal, Resilience, Coping

## Abstract

Early investigations of subjective well-being responses to the COVID-19 pandemic indicated average deterioration but also high variability related to vulnerability of population groups and pandemic phase. Thus, we aimed to gain new insights into the characteristics of certain groups and their differences in subjective well-being response patterns over time. First, we performed Latent Class Analyses with baseline survey data of 2,137 adults (mean age = 40.98, *SD* = 13.62) derived from the German CORONA HEALTH APP Study to identify subgroups showing similarity of a comprehensive set of 50 risk and protective factors. Next, we investigated the course of quality of life (QoL) as an indicator of subjective well-being grouped by the identified latent classes from July 2020 to July 2021 based on monthly and pandemic phase averaged follow-up survey data by means of Linear Mixed-Effects Regression Modeling. We identified 4 latent classes with distinct indicators and QoL trajectories (resilient, recovering, delayed, chronic) similar to previous evidence on responses to stressful life events. About 2 out of 5 people showed a resilient (i.e., relative stability) or recovering pattern (i.e., approaching pre-pandemic levels) over time. Absence of depressive symptoms, distress, needs or unhealthy behaviors and presence of adaptive coping, openness, good family climate and positive social experience were indicative of a resilient response pattern during the COVID-19 pandemic. The presented results add knowledge on how to adapt and enhance preparedness to future pandemic situations or similar societal crises by promoting adaptive coping, positive thinking and solidary strategies or timely low-threshold support offers.

**Supplementary Information:**

The online version contains supplementary material available at 10.1007/s12144-022-03628-4.

The ongoing COVID-19 pandemic represents a global crisis with consequences on the everyday lives and well-being of people from various ages, social and economic contexts (Vindegaard & Benros, 2020). On behalf of restriction measures to combat the spreading of the SARS-CoV-2 virus, people were challenged by changes in lifestyle, structure and routine (e.g., home office, home schooling, travel, nutrition and physical activity), insecurity and worries about health, financials and caregiving, over a considerable period of time (El Keshky et al., 2020; Prime et al., 2020). Thus, it has been discussed that people’s quality of life (QoL; i.e., subjective evaluations of and satisfaction with multiple life domains, most commonly comprising a physical, mental, social, and environmental dimension; Haas, 1999) is at risk of suffering and that there is the urgent need to address questions on how to mitigate maladaptive coping and long-lasting health consequences not only for survivors, families, and health care workers but the general population (Bryson, 2021; Ogueji et al., 2021).

A closer look at currently published results on peoples’ QoL suggests an average small to moderate decrease soon after the outbreak of the COVID-19 pandemic but also high variability between different samples and contexts (Herrera et al., 2021; Koivunen et al., 2022; Park et al., 2021; Rogers et al., 2021; van de Weijer et al., 2022). Results on the long-term development of QoL during the COVID-19 pandemic is fragmentary, so far. One study comparing the QoL during the initial phase of the COVID-19 pandemic with 8 months later observed a trend towards the approximation to average population levels for some domains (e.g., cognitive functioning, depressive symptoms) while others remained persistently worse (e.g., physical functioning, fatigue) (Rogers et al., 2021). This finding is supported by investigations on mental health outcomes indicating a temporary increase of certain psychopathological symptoms at the onset of the COVID-19 pandemic followed by a decrease to almost pre-pandemic levels for many people (Robinson et al., 2022; Vindegaard & Benros, 2020). Since the publication rate on COVID-19-related effects on the general population decreased after the first wave and most of the actual evidence refers to the initial phase of the pandemic, there is an ongoing need to further monitor the development and trend over time to identify phase-dependent or long-lasting effects (Mauz et al., 2022).

Notwithstanding the insufficient number of longitudinal population studies based on randomly drawn representative samples (Mauz et al., 2022; Vindegaard & Benros, 2020), there seems to be an agreement across studies on vulnerable groups that should be paid particular attention to. As proposed by the *Vulnerability-Stress Theory* (Rutter, 2006), the way people respond to stress is related to genetically determined, socially neglected or learned predispositions (i.e., *vulnerabilities*). In the face of a stressful life event, vulnerabilities can increase the risk of health deterioration and psychopathology (Rutter, 2006). However, it has to be considered that risk-enhancing factors do not occur isolated but interact with co-occurring risk-reducing factors (e.g., social support) and the strength of risk tends to vary across situations, life circumstances or stages (Lazarus, 1966). Hence, it is important to consider various potentially relevant factors at the same time. In the current pandemic situation, female sex, young adult age, pre-existing health conditions, pessimism, working in healthcare, job loss and financial insecurity, living alone or feelings of loneliness, COVID-19 infected relatives and health concerns were among the most frequently observed risk-enhancing factors for QoL limitations among people from various regions of the world (Algahtani et al., 2021; El Keshky et al., 2020; Epifanio et al., 2021; Gibson et al., 2021; Koivunen et al., 2022).

*Resilience*, on the contrary, describes the maintenance of subjective well-being and functioning over time (i.e., relatively stable trajectory) and as compared to others facing a comparable level of adversity (Bonanno, 2004). Despite inter-individual diversity in the presence of risk-enhancing and -reducing factors, resilience theory suggests shared mechanisms involved in the processing of a stressful life event (Mancini & Bonanno, 2009). The initial appraisal of the experience (e.g., as personally meaningful, threatening or overwhelming), availability of resources (e.g., social support or personal competencies) and coping strategy (e.g., emotion- or problem-focused) represent crucial mechanisms for adjustment and along the pathway to resilience (see *Transactional Stress and Coping Theory*, Lazarus & Folkman, 1984, and *Individual Differences Model*, Mancini & Bonanno, 2009). *Coping* has been conceptualized as both, a relatively stable (trait-like) tendency or style as well as a (situation-specific) dynamic process initiated by personally significant experience of harm or threat (Lazarus, 1993). One theoretically sound and empirically validated way to define and operationalize coping strategies is to differentiate between *emotion-focused* (i.e., regulation of negative emotional reactions), *problem-focused* (i.e., attempts to solve the problem), *meaning-focused* (i.e., positive reappraisal and acceptance) and *support-focused* (i.e., seeking instrumental and emotional support) coping (Folkman & Moskowitz, 2004, 2007). Specific situational characteristics of the COVID-19 pandemic and the corresponding restriction measures to combat the spreading of the SARS-CoV-2 virus are limitations of certainty, controllability and avoidability, which most probably affected the pursuit of coping responses and availability of resources. From stress response theory (Skinner & Zimmer-Gembeck, 2011) and investigations of comparable unavoidable stressful situations with limited control (e.g., chronic illness; Roesch & Weiner, 2001), we already know that certain coping efforts can be hampered and thus the risk of maladaptive coping increases. First investigations on coping responses to the COVID-19 pandemic moreover highlighted the level of perceived risk for self-protective or risk-taking behaviors (Gan & Fu, 2022; Motta Zanin et al., 2020). Additionally, availability of social resources such as face-to-face meetings and support systems (e.g., counselling) were also hampered due to stay-at-home-orders and people were challenged to compensate and were at higher risk of feelings of loneliness (Marroquín et al., 2020). Thus, it is important to enhance the understanding of reactivity and characteristics of resilient response patterns under the situational demanding circumstances of the COVID-19 pandemic.

Overall, there is mounting evidence suggesting that the majority of individuals respond in a *resilient* manner to stressful life events (35–65%; i.e., relatively stable high level, Bonanno et al., 2011). In addition to a resilient responding, the responses to stressful life events over time seem to follow a prototypical pattern of either *recovering* (15–25%; i.e., temporary decrease and gradual return), *chronic* (5–30%; i.e., persistently low level) or *delayed* (0–15%; i.e., steeper decrease and slower recovery) (Bonanno et al., 2011; Mancini & Bonanno, 2009). Some research also observed a response pattern that can be characterized as *improving* (i.e., temporary relief or lasting increase), for instance, after divorce or chronic illness (Bonanno et al., 2011; Mancini et al., 2011). While originating from studies addressing samples with a history of mental illness, the pattern seems to hold also true for well-being trajectories of the general population (Mancini et al., 2011). Large population-based studies yielded results that speak in favor of inter- as well as intra-individual heterogeneity in well-being responses to stressful life events with some people returning quickly to a *set-point*, as is a relatively stable personal baseline level (i.e., recovering pattern), while others do not (i.e., delayed pattern) and with some events raising the risk of permanent change (e.g., unemployment, financial deterioration) while others do not (e.g., divorce, job change) (Hentschel et al., 2017; Lucas, 2007; Luhmann et al., 2012). Therefore, in investigations on reactivity to stressful life events it is essential to consider both change and stability and to differentiate the trajectories over time by means of between- and within-person level comparison (Lucas, 2007; Mancini et al., 2011). Apart from the effects of type of event (Hentschel et al., 2017; Lucas, 2007; Luhmann et al., 2012) and controllability (Schwarzer & Schulz, 2003), it should be noted that the permanency (Hentschel et al., 2017) or repeated exposition (Luhmann & Eid, 2009) and possible experience of both positive and negative characteristics (Luhmann et al., 2012) are essential for investigations on stress responses in general and during the COVID-19 pandemic, in particular.

One promising method to identify population groups of similar health or well-being profiles, is a person-centered approach, such as the Latent Class Analysis (LCA; Eid, 2018). LCA particularly aims at detecting unobserved (latent) groups with shared characteristics and allows the simultaneous consideration of a large number of possible indicators (Collins & Lanza, 2009). This is particularly advantageous in view of the rather fragmentary evidence on risk factors during the COVID-19 pandemic. Accordingly, LCA has been increasingly applied in the context of crises and resilience research (Bonanno et al., 2011; Mancini & Bonanno, 2009; Mancini et al., 2011).

## Study rationale

The present study aimed to add insights to the previously incomplete and scarce knowledge of quality-of-life trajectories by looking at a one-year period marked by the COVID-19 pandemic and by considering an extensive selection of relevant situation-specific and general risk-increasing and risk-reducing factors. The leading research aim was to identify a resilient response pattern (i.e., stability of QoL over time) and distinct indicators of such a pattern. This information is highly valuable for public health protection, promotion and preparedness in case of the current COVID-19 pandemic and future similar situations. With reference to previous investigations of response patterns in the face of diverse stressful life events (Bonanno et al., 2011; Mancini et al., 2011) as well as in the past SARS (Bonanno et al., 2008) or the initial SARS-CoV-2 pandemic situation (Pierce et al., 2021; Shilton et al., 2021; Truskauskaite-Kuneviciene et al., 2021; Yalçın et al., 2022), we expected to find 3 to 5 different response patterns comprising one resilient subgroup.

## Methods

### Sample and Procedure

The present analyses are based on data derived from the CORONA HEALTH App Study collected from July 2020 to July 2021 (Beierle et al., 2021). The study addressed voluntary participants of at least 18 years of age recruited via the project partners’ institutional homepages and social media channels, mailing lists and media reports. We combined a cross-sectional (comprehensive baseline questionnaire) with a longitudinal design (reduced follow-up questionnaires), accompanied by a smartphone-delivered collection of data regarding communication app usage and the sensing of the GPS location at the time of answering the survey. After completing the baseline survey, participants were invited to answer follow-up surveys on a weekly basis. Participation could be cancelled, paused, and resumed at any time. With reference to the present analyses’ objectives (identification and tracing of QoL response patterns; see Study Rational), we considered self-report (survey) data, only. Moreover, the present analyses cover a data collection period of one year including phases of relaxation of the spreading of the SARS-CoV-2 virus after the second wave (July 2020 to October 2020, “pre lockdown”), intensification of infection rates and restriction measures during the third wave (November 2020 to March 2021, “lockdown”), followed by further relaxation of infection rates and restriction measures thereafter (April to July 2021, “post lockdown”) in Germany.

The original data base included 2,156 voluntary adult participants. Inspection of the plausibility of answers (e.g., correspondence between similar items), careless responding (straightlining and intraindividual response variability) and extreme outliers (Mahalanobis distance, Cook’s distance) led to the exclusion of 19 participants and a final sample size of 2,137 adults with an age range from 18 to 84 years (52.1% female, 47.3% male, 0.7% other; mean age = 40.98, *SD* = 13.62). 55% (*N* = 1,178; 55.0% female, 44.2% male, 0.7% other; mean age = 46.49, *SD* = 13.46) participated in the longitudinal part, with 2.3 times a month on average (*SD* = 1.30). The total number of answered questionnaires was *n* = 9,502 with a monthly average of *n* = 731 (*SD* = 291.71). Although the female to male ratio was relatively balanced, the sample was constituted of less old (60 years and older) and low educated (no school leaving certificate or primary education) participants. Table S1 in the Supplementary Materials presents more detailed sample characteristics for all variables under study.

## Measures

### Outcome

We used the EUROHIS-QOL-8-item index (Schmidt et al., 2005; e.g., satisfaction with personal relations, living conditions, ability to manage daily life) answered on a 5-point rating scale from 1 (very bad) to 5 (very good) to asses quality of life (see Table S1 in the Supplementary Materials). The internal consistency was α = 0.84.

### Indicators

As a standardized measure of psychopathological symptoms, we used the depression (9 items, e.g., little interest or pleasure in doing things; α = 0.90), general anxiety (7 items, e.g., not being able to stop or control worrying; α = 0.85) and short panic module (i.e., having a panic attack) of the Patient Health Questionnaire (PHQ-D; Löwe et al., 2002; Spitzer et al., 1999). We further used the standardized items of the PHQ-D (Löwe et al., 2002; Spitzer et al., 1999) psychosocial distress module (10 items, e.g., having no one to discuss problems with; α = 0.78) and experience of violence (e.g., being hit, kicked, or otherwise physically hurt). The 7-item version of the Insomnia Severity Index (ISI-7, Bastien et al., 2001; e.g., problems sleeping through; α = 0.91) was answered to indicate sleep problems. Loneliness was measured with the 3-item SOEP Loneliness Scale (LS-S, Richter & Weinhardt 2013; e.g., missing the company of others; α = 0.81). To measure the Big Five personality dimensions, we used the 10-item Big Five Inventory (BFI-10, Rammstedt et al., 2017; e.g.complete tasks thoroughly) comprising 2 items for each of the dimensions of openness, conscientiousness, extraversion, agreeableness, neuroticism (inter-item correlations were *r* = .82, 0.84, 0.83, 0.73, 0.75). As an indicator of situational coping strategy, we used the 28-item Brief-Cope Questionnaire (Carver, 1997) comprising 2 items for each of the 14 dimensions (e.g., denial, planning, humor). The dimensions were summarized to 4 latent factors (problem-focused, escape-avoidant-focused, meaning-focused and support-focused coping; α = 0.76, 0.72, 0.73, 0.83) in line with previous findings (Knoll et al., 2005) and as suggested by Carver (1997) based on Exploratory and Confirmatory Factor Analyses(Cohrdes et al., in revision). Subjective health, chronic conditions and health limitations were assessed via the first three items of the Mini European Health Module (MEHM, Cox et al., 2009; inter-item correlations were *r* = 0.43, 0.56). Stigmatization expectance and experience were assessed by 4 items adopted from the German version of the Inventory of Stigmatizing Experience (ISE, Schulze et al., 2009; e.g., observed or experienced teasing, bullying, or harassment because of COVID-19; inter-item correlations were *r* = 0.59, 0.51). Scoring procedures and cut-offs were followed according to the instructions of respective manuals (see Table S1 of the Supplementary Materials for further details).

In addition, several in-house developed items were included as indicators of COVID-19-related worries (infecting oneself or other people, becoming severely ill, lack of medical supplies) or job constraints (short-term work, closing of workplace or childcare facilities, quarantine), a current COVID-19 infection (oneself, relatives) and COVID-19-related death of relatives, financial loss, home-office, relevant sociodemographic characteristics (partnership status, household size, children, family climate, living conditions, working as healthcare professional), self-reported lifetime diagnosis of mental disorder, current psychotherapy, health behavior (physical activity, alcohol consumption), needs (e.g., instrumental or psychosocial support) and positive effects (e.g., societal cohesion or solidarity).

### Covariates

The participant’s age group (18–29, 30–44, 45–59, 60 + years), sex (female, male, other), and educational level (low = no school-leaving certificate or primary education [German “Hauptschulabschluss”], moderate = secondary education [German “Realschulabschluss” or “Fachabitur”], high = high school graduation [German “Abitur”]) were included as covariates in the analyses.

Table S1 in the Supplementary Materials gives a detailed overview of the variables under study.

## Statistical analyses

Latent Class Analyses (LCA) was used to identify unobserved (latent) groups based on similarity of response patterns in observed (manifest) variables under the assumption of statistical independency (Collins & Lanza, 2009). In preparation for LCA, we transformed metrical or multinomial variable values into binary categories coded at levels 1 and 2 (Linzer & Lewis, 2011). If applicable, we used prescribed cut-off values (e.g., psychopathological symptoms assessed with the PHQ-D) for dichotomizing the metric variable scores. Elsewise, metric variable scores or single item values were dichotomized based on 75% or 25% quantile bands of the distribution. For multinomial variable values we summarized answers based on theoretical and distribution-related appropriateness. For further details see the Measures section and Table S1 in the Supplementary Materials.

In the first set of analyses, we conducted subsequent LCA models by specifying 1 to 7 classes with several random starting values and 200 model estimate repetitions including the 50 indicators and 3 covariates. The analyses were conducted with the software R (R Core Team, 2021) and by using the poLCA (Linzer & Lewis, 2011) and tidyLPA (Rosenberg et al., 2018) packages. In poLCA, the expectation-maximization and Newton-Raphson algorithms are used to estimate the latent class model log-likelihood function. The following information criteria were used in order to assess the statistical model fit and optimal number of classes: Aikake´s Information Criteria (AIC), Bayesian Information Criteria (BIC), adjusted BIC, entropy and power based on Lo-Mendell-Rubin adjusted Likelihood Ratio Test (LMR-LRT) comparing k-1 classes. There were no missing variables due to forced choice response format.

In the second set of analyses, we conducted linear mixed-effects models (LME) with the lmerTest package (Kuznetsova et al., 2017). Thereby, we predicted the course of QoL scores (averaged by month and lockdown period) from the latent classes while taking repeated-measurements nested within persons as random effects into account. Linear mixed-effects modeling is appropriate for handling variation among the number or timing of observations (i.e., *unbalanced data*; Laird & Ware, 1982). In line with suggestions of higher statistical precision and avoidance of selection bias, we included all participants, even those with only baseline measurement, to estimate the changes (slopes) in the participant population (Thiébaut & Walker, 2008).

## Results

**Fig. 1:**
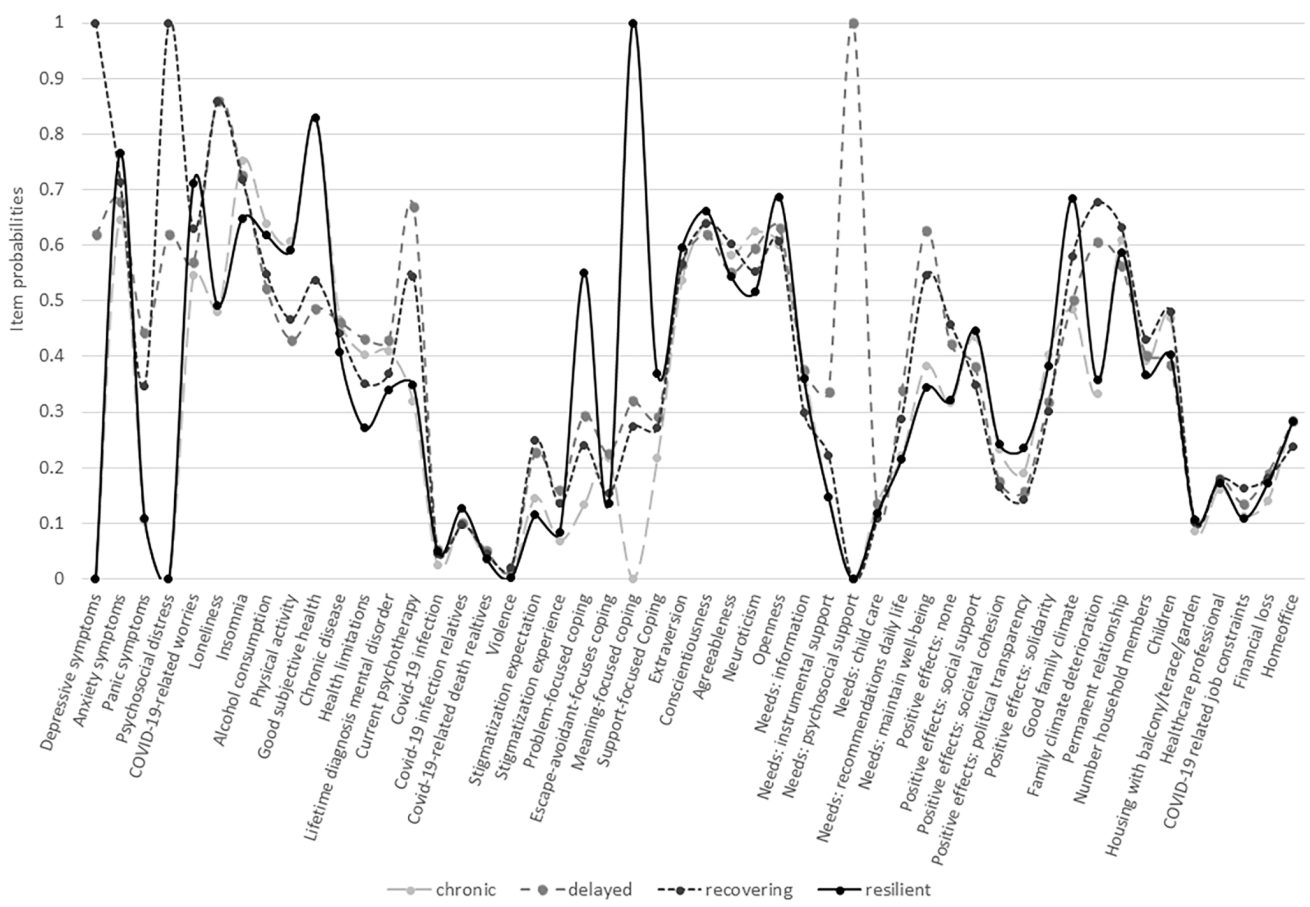
Item probabilities for each of the indicators under study (see the Supplementary Materials Table S2 in detail) grouped by four latent classes resulting from LCA

The results from LCA including a variety of 50 risk-enhancing and -reducing factors (see Fig. 1) and with the participant’s age, sex and educational level as covariates, suggested that a four and a five latent class solution fitted best the current data with regards to the information criteria presented in Table 1. On behalf of theory (Bonanno, 2004; Bonanno et al., 2011; Mancini & Bonanno, 2009), interpretability, and comparability to previous findings on latent classes in responses to the current and past pandemics (Bonanno et al., 2011; Mancini et al., 2011; Pierce et al., 2021; Shilton et al., 2021), we considered the four-class solution as optimal and focused in the following analyses on the four classes in more detail.

**Table 1:** Summary of Latent Class Analysis Information Criteria (*N* = 2,137)

# classes	MLL	AIC	BIC	aBIC	Entropy	LMR-LRT^1^
1	-173262.52	347521.10	350343.31	116525.09	--	--
2	-54319.42	108846.80	109435.84	109105.41	0.894	7661.74
3	-53391.99	107099.98	107994.80	107492.81	0.931	1854.86
**4**	**-52849.69**	**106123.39**	**107324.03**	**106650.48**	**0.937**	**1084.60**
5	-52437.69	105407.38	106913.84	106068.73	0.937	824.00
6	-52122.50	104885.01	106697.30	105680.63	0.916	630.38
7	-51822.60	104393.21	106511.33	105323.09	0.920	599.80

*Notes.* MLL = Maximum log-likelihood, AIC = Akaike information criterion, BIC = Bayesian information criterion, aBIC = adjusted Bayesian information criterion, LMR-LRT = Lo-Mendell-Rubin adjusted Likelihood Ratio Test. ^1^ LMR-LRT k(-1) Model comparisons were all significant at *p* < .001. The four-class solution was identified as optimal and highlighted in boldface

**Fig. 2:**
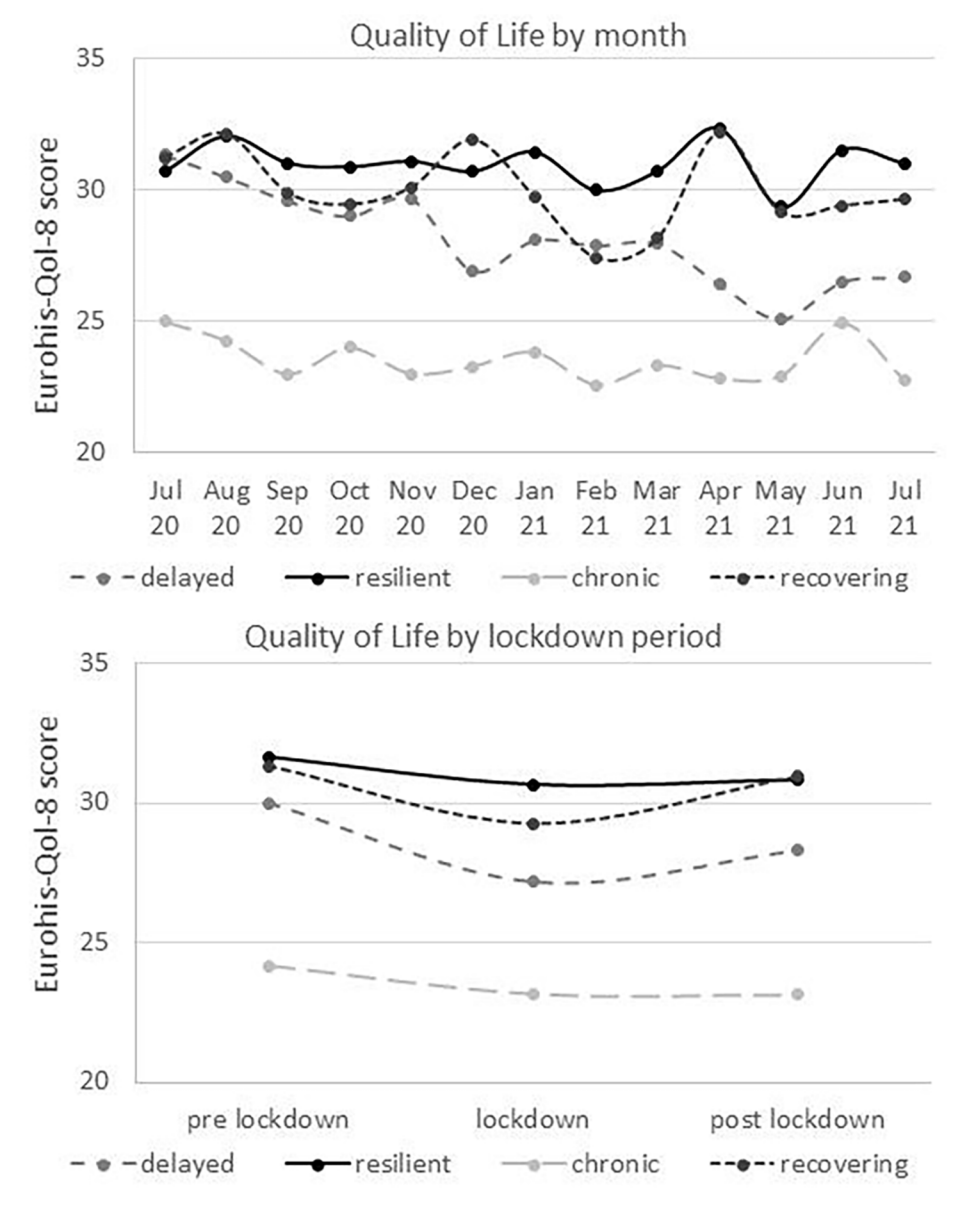
Subjective well-being trajectories during the COVID-19 pandemic, grouped by four latent classes as presented in Table 2 and Fig. 1, aggregated by month (above) and lockdown phase (below)

In consideration of the four latent trajectories as depicted in Fig. 2, the classes have been labelled as “resilient” (18.7%), “recovering” (20.6%), “delayed” (25.4%) and “chronic” (35.3%) based on prototypical patterns analogous to previous studies (Bonanno, 2005; Bonanno et al., 2008,2011; Mancini & Bonanno, 2009; Pierce et al., 2021; Shilton et al., 2021). Across time, QoL levels were the highest in the resilient class (*M* = 32.52, *SD* = 3.90), followed by the recovering (*M* = 30.82, *SD* = 4.41), delayed (*M* = 25.54, *SD* = 4.78) and chronic class (*M* = 23.17, *SD* = 5.21).

The resilient class is characterized by a low probability of depressive and panic symptoms or distress but moderate levels of anxiety symptoms and COVID-19-related worries. In addition, class members showed a high probability of good physical health and activity, family climate, conscientiousness and openness, meaning- and problem-focused coping, as well as positive experiences such as perceptions of social support and solidarity (see Fig. 1). In contrast, the chronic class is characterized by a high probability of insomnia, alcohol consumption, chronic disease, neuroticism, and a low probability of psychopathological symptoms or COVID-19-related worries, good family climate, meaning- and problem-focused coping. The recovering class is characterized by a high probability of depressive and anxiety symptoms, distress, perceived loneliness, stigmatization expectation, deterioration of family climate, having children, COVID-19-related job constraints and low probability of perceptions of positive effects (see Fig. 1, Table S2 of the Supplementary Materials). Characteristic for the delayed class was a comparatively high probability of panic symptoms, perceived loneliness, current psychotherapy, lifetime diagnosis of any mental disorder, health limitations, urgent need of psychosocial support and recommendations on how to maintain well-being in daily life, as well as a low probability of physical activity, good subjective health, perceiving positive effects and political transparency, in particular. Class membership differed only slightly according to the covariates with a tendency of young age, female sex and high education indicative for the resilient class. Table S2 in the Supplementary Materials presents all class probabilities in detail.

**Table 2:** Predicting Quality of Life by Four Latent Classes (Resilient, Recovering, Delayed, Chronic), Time aggregated in Months and Interactions based on Mixed-Effects Regression Modeling with the Participants as Random Effects Nested within Time (*N* = 2,137)

	QoL – M1		QoL – M2		QoL – M3		QoL – M4	
	*B (SE)*	*p*	*B (SE)*	*p*	*B (SE)*	*p*	*B (SE)*	*p*
Intercept	**31.92 (0.37)**	**< 0.001**	**31.31 (0.36)**	**< 0.001**	**32.15 (0.46)**	**< 0.001**	**24.28 (0.46)**	**< 0.001**
Resilient	*Ref*		0.61 (0.52)	0.236	-0.23 (0.59)	0.146	**7.64 (0.60)**	**< 0.001**
Recovering	-0.62 (0.52)	0.236	*Ref*		-0.84 (0.58)	0.695	**7.03 (0.59)**	**< 0.001**
Delayed	0.23 (0.59)	0.695	0.85 (0.58)	0.146	*Ref*		**7.87 (0.65)**	**< 0.001**
Chronic	**-7.64 (0.60)**	**< 0.001**	**-7.03 (0.69)**	**< 0.001**	**-7.88 (0.65)**	**< 0.001**	*Ref*	
Month	-0.14 (0.08)	0.067	**-0.47 (0.06)**	**< 0.001**	**-0.29 (0.08)**	**< 0.001**	-0.10 (0.07)	0.157
Resilient × Month	*Ref*		**0.34 (0.10)**	**< 0.001**	0.15 (0.11)	0.189	-0.03 (0.11)	0.742
Recovering × Month	**-0.33 (0.10)**	**< 0.001**	*Ref*		**-0.19 (0.09)**	**0.047**	-**0.37 (0.10)**	**< 0.001**
Delayed × Month	-0.15 (0.11)	0.107	**-0.19 (0.09)**	**0.047**	*Ref*		-0.18 (0.11)	0.100
Chronic × Month	0.03 (0.11)	0.741	-**0.37 (0.10)**	**< 0.001**	0.18 (0.11)	0.100	*Ref*	
Random effect ID:Time *Var* = 18.00, *SD* = 4.24								

*Notes.* QoL = Quality of Life, M = Model, *B* = Unstandardized beta coefficient, *SE* = Standard error, Ref = Reference category, Var = Variance, *SD* = Standard deviation. M1 = Resilient as reference category, M2 = Recovering as reference category, M3 = Delayed as reference category, M4 = Chronic as reference category. Boldface indicates significant results at *p* < .05

Tables 2 and 3 present the results from four LME models with each of the latent classes as reference category and QoL responses aggregated within-persons on a monthly basis as well as separated by lockdown period. The results suggest a significant main effect of the chronic class with significantly lower QoL as compared to the other classes. In addition, grouping by lockdown period lead to significant QoL differences of the recovering class compared to the resilient, chronic and delayed class (Table 3). While QoL levels remained relatively constant over time for the resilient and chronic class, we found a significant main effect of time (see Tables 2 and 3) as well as interactions with time for the recovering and delayed class, as displayed in Fig. 2. The average QoL level of the recovering class declined from pre-lockdown to lockdown and approximated the baseline level thereafter (post-lockdown); the delayed class showed a steeper decrease of QoL during the lockdown phase followed by a slower increase during the post-lockdown phase.

**Table 3:** Predicting Quality of Life by Four Latent Classes (Resilient, Recovering, Delayed, Chronic), Time aggregated in Lockdown Phases and Interactions based on Mixed-Effects Regression Modeling with the Participants as Random Effects Nested within Time (*N* = 2,137)

	QoL – M1		QoL – M2		QoL – M3		QoL – M4	
	*B (SE)*	*p*	*B (SE)*	*p*	*B (SE)*	*p*	*B (SE)*	*p*
Intercept	**30.57(0.55)**	**< 0.001**	**27.18 (0.36)**	**< 0.001**	**29.24(0.57)**	**< 0.001**	**23.17 (0.47)**	**< 0.001**
Resilient	*Ref*		**3.39(0.66)**	**< 0.001**	1.33(0.79)	0.095	**7.41(0.73)**	**< 0.001**
Recovering	**-3.39(0.65)**	**< 0.001**	*Ref*		**-2.06(0.68)**	**0.002**	**4.01(0.59)**	**< 0.001**
Delayed	-1.33(0.79)	0.095	**2.06(0.67)**	**0.002**	*Ref*		**6.07(0.74)**	**< 0.001**
Chronic	**-7.41(0.72)**	**< 0.001**	**-4.01(0.59)**	**< 0.001**	**-6.07(0.74)**	**< 0.001**	*Ref*	
Lockdown vs. pre	1.05(0.62)	0.089	**2.79(0.44)**	**< 0.001**	**2.07(0.84)**	**0.002**	0.97(0.57)	0.093
Lockdown vs. post	0.25(0.82)	0.762	**1.10(0.55)**	**0.048**	**1.70(0.84)**	**0.043**	-0.04(0.76)	0.961
Resilient Lockdown × pre	*Ref*		**-1.73(0.76)**	**0.021**	-1.02(0.91)	0.262	0.08(0.84)	0.926
Recovering Lockdown × pre	**1.74(0.75)**	**0.021**	*Ref*		0.71 (0.80)	0.368	**1.81(0.73)**	**0.012**
Delayed Lockdown × pre	1.02(0.91)	0.065	-0.72(0.80)	0.368	*Ref*		1.10(0.87)	0.213
Chronic Lockdown × pre	-0.08(0.84)	0.926	**-1.82(0.73)**	**0.012**	-1.10(0.88)	0.213	*Ref*	
Resilient Lockdown × post	*Ref*		-0.85(0.99)	0.392	-1.45(1.17)	0.217	0.29(1.12)	0.798
Recovering Lockdown × post	0.85(0.99)	0.392	*Ref*		-0.60(1.01)	0.553	1.14(0.94)	0.229
Delayed Lockdown × pre	1.45(1.17)	0.217	0.60(1.01)	0.553	*Ref*		1.73(1.13)	0.125
Chronic Lockdown × post	-0.29(1.12)	0.798	-1.14(0.95)	0.229	-1.73(1.13)	0.125	*Ref*	
Random effect ID:Time *Var* = 20.97, *SD* = 4.58								

*Notes.* QoL = Quality of Life, M = Model, *B* = Unstandardized beta coefficient, *SE* = Standard error, Ref = Reference category, Var = Variance, *SD* = Standard deviation. M1 = Resilient as reference category, M2 = Recovering as reference category, M3 = Delayed as reference category, M4 = Chronic as reference category. Boldface indicates significant results at *p* < .05

## Discussion

This study aimed to contribute to the understanding of differences and changes of adult QoL over a period of one year during the COVID-19 pandemic in Germany and with regards to substantial risk-enhancing or -reducing factors. Of particular interest was the question as to which factors are predictive of a resilient response pattern (i.e., maintaining relatively high levels of QoL) to derive recommendations for better preparedness and universal prevention measures.

In general, the actual QoL was slightly lower as compared to German adult norm values from 2004: the average across items was 3.61 (*SD* = 0.74) vs. 4.08 (*SD* = 0.49) (Schmidt et al., 2005) and the raw score was 28.91 (*SD* = 5.90) vs. 30.88 (*SD* = 4.59) (Brähler et al., 2007). However, the extensive period of 17 years between the two measurement points and non-representativeness of the present sample hinder interpretability of comparison and conclusion about mean-level difference related to the COVID-19 pandemic. By taking also other research into account (van de Weijer et al., 2022), the overall results suggest that the COVID-19 pandemic may not manifest in substantial average QoL change or affect adult QoL levels equally. However, there certainly seem to be subgroups vulnerable to QoL deterioration. In fact, we identified four latent classes (resilient, recovering, delayed and chronic) with distinct QoL patterns in accordance with evidence on prototypical responses to stressful life events from previous research (Bonanno et al., 2011; Mancini et al., 2011) as well as from the initial phase of the COVID-19 pandemic (Pierce et al., 2021; Shilton et al., 2021). Two of these classes, namely the resilient and chronic subgroup, showed relatively stable trajectories over a period of one year on a rather high or low QoL level, respectively. In contrast, the other two classes, showed instability of QoL levels over time indicating adjustment processing in response to the situational demands of the COVID-19 pandemic. Whereas the QoL levels decreased at first in both classes, the recovering class returned almost to baseline level over time and the delayed class showed a steeper decrease and slower increase not reaching the initial baseline level. Thus, the delayed class can be seen as particularly vulnerable to QoL deterioration during the COVID-19 pandemic.

In line with previous evidence on COVID-19-related risk factors (Algahtani et al., 2021; El Keshky et al., 2020; Epifanio et al., 2021; Gibson et al., 2021; Koivunen et al., 2022), we found that people with pre-existing health conditions (lifetime diagnosis of mental disorder, chronic illness, functional limitations) or currently undergoing psychotherapeutic treatment, a rather pessimistic view of the situation (no positive experiences, expecting stigmatization), COVID-19-related job constraints or financial loss, living alone or feeling lonely, had the highest probability of QoL limitations and fell either into the recovering, delayed or chronic class. In contrast to previous findings, we could not find any signs of relatedness between class membership with working in healthcare (Papoutsi et al., 2020) or the occurrence of COVID-19 infections among relatives (Beck et al., 2021), which may be due to differences in health-care systems, amount of COVID-19-related hospitalizations, mortality rate or sampling bias (e.g., under-representation or lack of certain job information such as working in intensive or outpatient care) (Skoda et al., 2020).

Additionally, we observed a high probability of symptoms of depression, anxiety and psychosocial distress in the recovering class. Thus, the results reflect a frequently observed trend of temporarily enhanced psychopathological symptoms that seemed to level off at a certain stage of the COVID-19 pandemic (Mauz et al., 2022; Robinson et al., 2022; Vindegaard & Benros, 2020). The recovering class moreover included people most likely to have children, experiencing deterioration of family climate and COVID-19-related job constraints. Thereby, the recovering class can be seen as representative for a large proportion of families struggling with COVID-19-related losses of income, access to resources, daily routines and planned activities, as well as work-from-home arrangements in combination with childcare (Lee et al., 2020; Prime et al., 2020). In contrast, members of the chronic class showed less signs of COVID-19-related symptoms or worries but reported more unhealthy lifestyle behaviors (insomnia, alcohol consumption), high neuroticism and maladaptive coping in addition to chronic illness. While chronic illness can be seen as one of the most comprehensible indicators of reduced QoL irrespective of situational context (Megari, 2013), there is also evidence on interrelatedness between the other indicators. Neuroticism has been related to a relatively stable trend of maladaptive responding to stressors (i.e., high negative affective reactivity), risky health behaviors, higher risk of mental and physical illness, and mortality (Lahey, 2009). Overall, coping strategy and other personal or social resources (personality, family climate) in this research became apparent as central indicators differentiating between groups of people showing diverse QoL trajectories, as proposed by resilience and stress response theories (Lazarus & Folkman, 1984; Mancini & Bonanno, 2009). Most obviously, meaning-focused coping (e.g., positive reframing and acceptance) was predictive of resilient responding, underscoring previous assumptions on its increased importance particularly in uncontrollable stressful situations (Folkman & Moskowitz, 2007) and during the COVID-19 pandemic in particular (Shamblaw et al., 2021). Relatively high perceptions of solidarity and seldom reports of stigmatization expectation or experience due to a COVID-19 infection were among the most positive experiences during the COVID-19 pandemic were. Both applies to the members of the resilient class.

However, the proportion of resilient individuals in the present study was relatively low as compared to other studies during the COVID-19 pandemic (e.g., in the UK (Pierce et al., 2021), USA and Israel (Shilton et al., 2021), Lithuania and Germany (Truskauskaite-Kuneviciene et al., 2021) or Turkey (Yalçın et al., 2022)). One possible explanation is that the other studies focused on mental health outcomes as indicators of resilient responding (Pierce et al., 2021; Shilton et al., 2021; Truskauskaite-Kuneviciene et al., 2021; Yalçın et al., 2022) while the present research investigated QoL, closely related to other well-being or health measures but representing a distinct dimensional construct (Keyes, 2014). The risk of developing a manifest mental illness during the challenges related to the COVID-19 pandemic may be not as high as the risk of deteriorations of subjective well-being. Future research will have to elaborate on that in more detail.

Summarizing the amount of resilient and recovering response patterns leads to the conclusion that the highest proportion but not even half of the people showed a high probability of adjustment to the challenges of the COVID-19 pandemic while about one fourth was at risk of long-lasting QoL deterioration (delayed class). By taking also into consideration that a considerable proportion of participants fell into the chronic class with comparatively low QoL and high psychopathological symptoms at the same time, highlight the need of both mental illness prevention and QoL promotion in line with the conceptualization of mental health as a *dual continuum* and *complete state* (i.e., differentiating between absence of illness and presence of negative well-being or functioning, Keyes 2014). The present findings imply that healthy behaviors (e.g., regular physical activity) and adaptive stress reactivity competencies (e.g., emotional stability as opposed to neuroticism, positive reframing as opposed to denial) may be a good starting point for universal prevention and promotion at the population level. Importantly, the burden of the delayed class can be seen as particularly worrisome with respects to the demands related to the COVID-19 pandemic. People in this class reported unmet needs such as psychosocial support and showed the steepest decline over the period of one year even though receiving psychotherapy to a large extent. Accordingly, people in the delayed class seem to require more or other than the already established prevention and protection efforts to enhance (faster) recovery or resilience.

In consideration of moderate anxiety and COVID-19-related worries even in the resilient class suggests that individuals of each of the four classes have to deal with certain risk factors but the resilient class differentiates from the others by showing maintenance of QoL despite comparable level of adversity, as suggested by resilience theory (Rutter, 2006). It is to be assumed that a certain level of anxiety and worry is a normal reaction and concurrently present risk-reducing factors help compensate and maintain QoL despite certain risk-enhancing factors (e.g., COVID-19-related worries and anxiety symptoms). Other findings underscore this assumption by showing how individuals during the COVID-19 pandemic mobilized resources to compensate needs and maintain their QoL (Herrera et al., 2021). However, these post-hoc explanations were not tested and need further investigation on interaction effects.

## Limitations

This study has some limitations that should be considered for interpreting the results. First, the data base was a convenience sample not allowing any generalization of findings to the German population. More precisely, the sample was unbalanced with regard to the educational level and age, comprising a greater amount of people with higher education and from young to middle adulthood. Therefore, the educational level and age were consistently included as covariates in the present analyses. Second, the data collection was undertaken by a smartphone app, thereby limiting the sample to participants who had access and were proficient with smartphones. Third, despite the longitudinal design and advantage of tracing QoL levels over time, this research lacks pre-pandemic QoL levels. Thus, conclusions are limited to the pandemic phase and do not allow continuous pre- to post comparisons. Although the data were collected during the COVID-19 pandemic, recall bias cannot be ruled out either. Furthermore, the interfaces for Android users and iOS users never look the same, which can generally create an information bias.

## Conclusion

The results highlight the central need of adaptive coping competency, positive social experience such as solidarity as well as low-threshold psychosocial support and prompt easily accessible recommendations on how to maintain well-being during a societal crisis such as the COVID-19 pandemic. Public health measures should be addressed particularly to people showing considerable QoL deterioration without returning to baseline levels (delayed and chronic response pattern) in both a preventive and promotive matter to enhance preparedness and resilience.

## Electronic supplementary material

Below is the link to the electronic supplementary material.

Supplementary Material 1

## Data Availability

The data presented in this study are available on request from the corresponding author. The data are not publicly available because participants’ informed consent did not cover public deposition of data.
