# Supplementary material for: Adult quality of life patterns and trajectories during the COVID-19 pandemic in Germany
Source: Curr Psychol. 2022 Sep 30:1–13. Online ahead of print. doi: 10.1007/s12144-022-03628-4 (PMC9523181; doi:10.1007/s12144-022-03628-4)
Supplement: Supplementary file 1 — Supplementary Material 1 [file 12144_2022_3628_MOESM1_ESM.docx]

**Supplementary Materials**

**Manuscript Title: Quality of Life Patterns and Trajectories in German Adults During the COVID-19 Pandemic**

*Table S1.*

Summary statistics and data transformation procedure for each variable under study for *N*=2137 participating adults living in Germany (July 2020-July 2021)

| **Construct, variable values** | **# Items** | **Instrument** | ***M* (*SD*)** | **Min/Max,**  **value labels** | **Dichotomization** | ***N* (%)** |
| --- | --- | --- | --- | --- | --- | --- |
| **Quality of Life (Outcome)** | 8 | EUROHIS-QOL-8  [1] | 28.91 (5.90) | 1/5  1=very bad, to 5=very good | -- | -- |
| **Age (Covariate)^1^** | 1 | In-house | 40.98 (13.62) | 18/78 | -- |  |
| 18-29 years |  |  |  |  |  | 494 (23.1%) |
| 30-44 years |  |  |  |  |  | 828 (38.8%) |
| 45-59 years |  |  |  |  |  | 590 (27.6%) |
| 60+ years |  |  |  |  |  | 225 (10.5%) |
| **Sex (Covariate) ^1^** | 1 | In-house | -- | 0/2 | -- |  |
| Female (0) |  |  |  |  |  | 1113 (52.1%) |
| Male (1) |  |  |  |  |  | 1009 (47.2%) |
| Other (2) |  |  |  |  |  | 15 (0.7%) |
| **Education (Covariate) ^1 2^** | 1 | CASMIN [2] | -- | 0=no school-leaving certificate or primary education  1=secondary education  2=high school graduation | -- |  |
| Low |  |  |  |  |  | 146 (6.9%) |
| Moderate |  |  |  |  |  | 842 (39.5%) |
| High |  |  |  |  |  | 1141 (53.6%) |
| **Depressive symptoms** | 9 | PHQ-9 from  PHQ-D [3] | 8.44 (6.22) | 0/3  0=never, to  3=almost every day | (1) Sumscore >=10 |  |
| Mild to severe (1) |  |  |  |  |  | 777 (36.4%) |
| No (0) |  |  |  |  |  | 1360 (63.6%) |
| **Anxiety symptoms** | 7 | GAD-7 from PHQ-D [3] | 7.53 (4.93) | 0/3  0=never, to  3=almost every day | (1) Sumscore >=10 |  |
| Mild to severe (1) |  |  |  |  |  | 667 (31.2%) |
| No (0) |  |  |  |  |  | 1470 (68.8%) |
| **Panic symptoms** | 1 | PHQ-D [3] |  | 0/1 | -- |  |
| Yes (1) |  |  |  |  |  | 497 (23.3%) |
| No (0) |  |  |  |  |  | 1640 (76.7%) |
| **Psychosocial distress** | 10 | PHQ-D [3] | 7.83 (4.64) | 0/2  0=no,  1=mild,  2=severe | (1) Sumscore >=10 |  |
| Mild to severe (1) |  |  |  |  |  | 755 (35.3%) |
| No (0) |  |  |  |  |  | 1382 (64.7%) |
| **Covid-19 related worries (e.g., infect others, lack of medical supplies)** | 4 | In-house | 4.02 (2.35) | 0/2  0=no,  1=mild,  2=severe | (1) >=75% quartile |  |
| Mild to severe (1) |  |  |  |  |  | 1504 (60.4%) |
| No (0) |  |  |  |  |  | 633 (29.6%) |
| **Loneliness** | 3 | LS-S [4] | 9.01 (3.12) | 3/15  1=very often, to 5=never | (1) >=75% quartile |  |
| Very often to often (1) |  |  |  |  |  | 691 (32.3%) |
| Sometimes to never (0) |  |  |  |  |  | 1446 (67.7%) |
| **Insomnia** |  | ISI-7 [5] |  | 0/28 | (1) Sumscore >=15 |  |
| Moderate to severe (1) |  |  | 9.32 (6.39) |  |  | 475 (22.2%) |
| None to mild (0) |  |  |  |  |  | 1662 (77.8%) |
| **Alcohol consumption (past week)** |  | In-house | 1.10 (1.17) | 0/3  0=never, to  3=6 to 7 times | (1) >=75% quartile |  |
| >= 2 times a week (1) |  |  |  |  |  | 696 (32.5%) |
| Never or once (0) |  |  |  |  |  | 1442 (67.5%) |
| **Physical activity (past week)** |  | In-house | 2.54 (1.37) | 1/5  1=none, to  5=more than 4 hrs | (1) >=75% quartile |  |
| Regulalrly, >= 2 hrs (1) |  |  |  |  |  | 579 (27.1%) |
| None or < 1 hr (0) |  |  |  |  |  | 1558 (72.9%) |
| **Subjective health status** |  | MEHM [6] | 2.18 (0.85) | 1/5  1=very good, to 5=very bad | (1) <=25% quartile |  |
| Very good to good (1) |  |  |  |  |  | 1475 (69.0%) |
| Moderate to bad (0) |  |  |  |  |  | 662  (31.0%) |
| **Chronic disease^1^** |  | MEHM [6] |  | 0/1 | -- |  |
| Yes (1) |  |  |  |  |  | 961  (44.9%) |
| No (0) |  |  |  |  |  | 1176  (55.1%) |
| **Health limitations^1^** |  | MEHM [6] |  | 0/3  0=no,  1=mild,  2=severe | 1. >=1 |  |
| Yes, mild to severe (1) |  |  |  |  |  | 805  (37.7%) |
| No (0) |  |  |  |  |  | 1332  (62.3%) |
| **Self-reported lifetime diagnosis any mental disorder^1^** |  | In-house |  |  |  |  |
| Yes (1) |  |  |  |  |  | 842 (39.4%) |
| No (0) |  |  |  |  |  | 1295 (60.6%) |
| **Current psychotherapy** |  | In-house |  | 0/1 | -- |  |
| Yes (1) |  |  |  |  |  | 235 (11.0%) |
| No (0) |  |  |  |  |  | 1902  (89.0%) |
| **COVID-19 infection** | 1 | In-house |  | 0/1 | -- |  |
| Yes (1) |  |  |  |  |  | 85  (4.0%) |
| No (0) |  |  |  |  |  | 2052 (96.0%) |
| **COVID-19 infection of relatives** | 1 | In-house |  | 0/1 | -- |  |
| Yes (1) |  |  |  |  |  | 221 (10.3%) |
| No (0) |  |  |  |  |  | 1916 (89.7%) |
| **COVID-19 related death of relatives** | 1 | In-house |  | 0/1 | -- |  |
| Yes (1) |  |  |  |  |  | 86 (3.0%) |
| No (0) |  |  |  |  |  | 2051 (96.0%) |
| **Violence** (past week) | 1 | PHQ-D [3] |  | 0/1 |  |  |
| Yes (1) |  |  |  |  |  | 22 (1.0%) |
| No (0) |  |  |  |  |  | 2115 (99.0%) |
| **Stigmatization expectation^1^** | 2 | Adaption of ISE [7] | 3.18 (0.87) | 2/10  1=always, to  5=never | -- |  |
| Yes, sometimes to always (1) |  |  |  |  |  | 641 (30.0%) |
| Never or seldom (0) |  |  |  |  |  | 1496 (70.0%) |
| **Stigmatization experience^1^** | 2 | Adaption of ISE [7] |  | 0/1 | -- |  |
| Yes (1) |  |  |  |  |  | 226 (10.6%) |
| No (0) |  |  |  |  |  | 1911 (89.4%) |
| **Coping^1^** | 28 | Brief-COPE [8] |  | 1/4  1=not at all, to  4=very much |  |  |
| Problem-focused |  |  | 2.49 (0.63) |  | (1) >=75% quartile | 560 (26.2%) |
| High (1) |  |  |  |  |  |  |
| Moderate to low (0) |  |  |  |  |  |  |
| Escape-avoidant-focused |  |  | 1.41 (0.48) |  | (1) >=75% quartile | 410 (19.2%) |
| High (1) |  |  |  |  |  |  |
| Moderate to low (0) |  |  |  |  |  |  |
| Meaning-focused |  |  | 2.42 (0.62) |  | (1) >=75% quartile | 637 (29.8%) |
| High (1) |  |  |  |  |  |  |
| Moderate to low (0) |  |  |  |  |  |  |
| Support-focused |  |  | 2.00 (0.72) |  | (1) >=75% quartile | 578 (27.0%) |
| High (1) |  |  |  |  |  |  |
| Moderate to low (0) |  |  |  |  |  |  |
| **Personality^1^** | 10 | BFI-10 [9] |  | 1/5  1=not at all, to 5=totally |  |  |
| Extraversion |  |  | 3.29 (0.96) |  | (1) >=75% quartile | 732 (34.3%) |
| High (1) |  |  |  |  |  |  |
| Moderate to low (0) |  |  |  |  |  |  |
| Openess |  |  | 3.81 (0.87) |  | (1) >=75% quartile | 677 (31.7%) |
| High (1) |  |  |  |  |  |  |
| Moderate to low (0) |  |  |  |  |  |  |
| **Conscientiousness** |  |  | 3.83 (0.73) |  | (1) >=75% quartile | 561 (26.3%) |
| High (1) |  |  |  |  |  |  |
| Moderate to low (0) |  |  |  |  |  |  |
| Agreeableness |  |  | 3.31 (0.77) |  | (1) >=75% quartile | 684 (32.0%) |
| High (1) |  |  |  |  |  |  |
| Moderate to low (0) |  |  |  |  |  |  |
| Neuroticism |  |  | 3.45 (0.88) |  | (1) >=75% quartile | 850 (39.8%) |
| High (1) |  |  |  |  |  |  |
| Moderate to low (0) |  |  |  |  |  |  |
| **Family climate** | 1 | In-house | 2.54 (0.89) | 0/4  0=very bad, to  4=very good | (1) >=75% quartile |  |
| Good to very good (1) |  |  |  |  |  | 978 (45.8%) |
| Moderate to very bad (0) |  |  |  |  |  | 1159 (54.2%) |
| **Family climate change^1^** | 1 | In-house | 0.61 (0.63) | 0/2  0=declined, 1=constant, 2=improved |  |  |
| Improved or constant (1) |  |  |  |  |  | 1129 (52.8%) |
| Declined (0) |  |  |  |  |  | 1008 (47.2%) |
| **Needs** | 6 |  | In-house |  |  |  |
| Information |  |  |  | 0/1 | -- |  |
| Yes (1) |  |  |  |  |  | 1373 (64.2%) |
| No (0) |  |  |  |  |  | 764 (35.8%) |
| Instrumental support |  |  |  |  | -- |  |
| Yes (1) |  |  |  | 0/1 |  | 1698 (79.5%) |
| No (0) |  |  |  |  |  | 439 (20.5%) |
| Psychosocial support |  |  |  | 0/1 | -- |  |
| Yes (1) |  |  |  |  |  | 1658 (77.6%) |
| No (0) |  |  |  |  |  | 479 (22.4%) |
| Child care |  |  |  | 0/1 | -- |  |
| Yes (1) |  |  |  |  |  | 1820 (85.2%) |
| No (0) |  |  |  |  |  | 317 (14.8%) |
| Recommendations daily life |  |  |  | 0/1 | -- |  |
| Yes (1) |  |  |  |  |  | 1580 (73.9%) |
| No (0) |  |  |  |  |  | 557 (26.1%) |
| Recommendations maintain well-being |  |  |  | 0/1 | -- |  |
| Yes (1) |  |  |  |  |  | 1142 (53.4%) |
| No (0) |  |  |  |  |  | 995 (46.6%) |
| **Postive effects** | 5 | In-house |  |  |  |  |
| None |  |  |  | 0/1 | -- |  |
| Yes (1) |  |  |  |  |  | 1343 (62.8%) |
| No (0) |  |  |  |  |  | 794 (37.2%) |
| Social support |  |  |  | 0/1 | -- |  |
| Yes (1) |  |  |  |  |  | 1268 (59.3%) |
| No (0) |  |  |  |  |  | 869 (40.7%) |
| Societal cohesion |  |  |  | 0/1 | -- |  |
| Yes (1) |  |  |  |  |  | 1694 (79.3%) |
| No (0) |  |  |  |  |  | 443 (20.7%) |
| Political transparency |  |  |  | 0/1 | -- |  |
| Yes (1) |  |  |  |  |  | 1752 (82.0%) |
| No (0) |  |  |  |  |  | 385 (18.0%) |
| Solidarity |  |  |  | 0/1 | -- |  |
| Yes (1) |  |  |  |  |  | 1371 (64.2%) |
| No (0) |  |  |  |  |  | 766 (35.8%) |
| **Permanent relationship** | 1 | In-house |  | 0/1 |  |  |
| Yes (1) |  |  |  |  |  | 1282 (60.0%) |
| No (0) |  |  |  |  |  | 855 (40.0%) |
| **Household size^1^** | 1 | In-house | 2.49 (1.38) | 1/12 | 1. >=75% quartile |  |
| >= 3 members (1) |  |  |  |  |  | 851 (39.8%) |
| 1-2 members (0) |  |  |  |  |  | 1286 (60.2%) |
| **Children^1^** | 1 | In-house | 0.84 (1.11) | 0/5 | 1. >=1 |  |
| Yes (1) |  |  |  |  |  | 943 (44.1%) |
| No (0) |  |  |  |  |  | 1194 (55.9%) |
| **Housing with balcony, terrace or garden** | 1 | In-house |  | 0/1 | -- |  |
| Yes (1) |  |  |  |  |  | 1771 (82.9%) |
| No (0) |  |  |  |  |  | 366 (17.1%) |
| **Healthcare professional** | 1 | In-house |  | 0/1 | -- |  |
| Yes (1) |  |  |  |  |  | 366 (17.1%) |
| No (0) |  |  |  |  |  | 1771 (82.8%) |
| **COVID-19-related job constraints (e.g., closing childcare facilities, short-term work)** | 5 | In-house |  | 0/1 | -- |  |
| Yes (1) |  |  |  |  |  | 274 (12.8%) |
| No (0) |  |  |  |  |  | 1863 (87.2%) |
| **Financial loss** | 1 | In-house |  | 0/1 | -- |  |
| Yes, slight to severe (1) |  |  |  |  |  | 467 (21.8%) |
| No (0) |  |  |  |  |  | 1670 (83.4%) |
| **Home office** | 1 | In-house |  | 0/1 | -- |  |
| Yes, full- or part-time (1) |  |  |  |  |  | 1550 (65.1%) |
| No (0) |  |  |  |  |  | 587 (34.9%) |

*Notes.* **^1^**Measured at baseline only (else at each measurement occasion). Mean values for repeatedly measured continuous variables were within-person centered and for categorical variables we used the within-person mode to generate overall measurement occasion summary statistics. ^2^Eight missing values due to graduation obtained outside of Germany.

*Table S2.*

Latent class proportions for covariates and probabilities for indicators (see also Figure 1 in the main text)

|  | **Chronic (35.3%)** | **Delayed (25.4%)** | **Recovering (20.6%)** | **Resilient (18.7%)** |
| --- | --- | --- | --- | --- |
| **Covariates** |  |  |  |  |
| Age: 18-29 y. | 169 (34.2%) | 130 (26.3%) | 97 (19.6%) | 98 (19.8%) |
| Age: 30-44 y. | 335 (39.5%) | 166 (19.6%) | 178 (21.0%) | 149 (17.6%) |
| Age: 45-59 y. | 238 (40.3%) | 131 (22.2%) | 135 (22.9%) | 86 (14.6%) |
| Age: 60 + y. | 100 (44.4%) | 52 (23.1%) | 48 (21.3%) | 25 (11.1%) |
| Sex: female | 430 (38.6%) | 253 (22.7%) | 228 (20.5%) | 202 (18.2%) |
| Sex: male | 405 (40.1%) | 221 (21.9%) | 228 (22.6%) | 155 (15.4%) |
| Sex: diverse | 7 (4.7%) | 5 (0.3%) | 2 (13.3%) | 1 (6.7%) |
| Education: low | 62 (44.9%) | 41 (29.7%) | 22 (25.9%) | 12 (8.7%) |
| Education: moderate | 349 (41.4%) | 173 (20.5%) | 193 (22.9%) | 127 (15.1%) |
| Education: high | 424 (37.2%) | 261 (22.9%) | 239 (20.9%) | 217 (19.0%) |
| **Indicators** |  |  |  |  |
| Depressive symptoms | 0.00 | 0.62 | 1.00 | 0.00 |
| Anxiety symptoms | 0.65 | 0.68 | 0.77 | 0.71 |
| Panic symptoms | 0.10 | 0.44 | 0.35 | 0.11 |
| Psychosocial distress | 0.00 | 0.62 | 1.00 | 0.00 |
| COVID-19-related worries | 0.55 | 0.57 | 0.63 | 0.71 |
| Loneliness | 0.48 | 0.86 | 0.86 | 0.49 |
| Sleep disturbance | 0.75 | 0.73 | 0.72 | 0.65 |
| Alcohol consumption | 0.64 | 0.52 | 0.55 | 0.62 |
| Physical activity | 0.61 | 0.43 | 0.47 | 0.59 |
| Good subjective health | 0.83 | 0.49 | 0.54 | 0.83 |
| Chronic disease | 0.47 | 0.46 | 0.44 | 0.41 |
| Health limitations | 0.40 | 0.43 | 0.35 | 0.27 |
| Self-reported lifetime diagnosis any mental disorder | 0.41 | 0.43 | 0.37 | 0.34 |
| Current psychotherapy | 0.32 | 0.67 | 0.54 | 0.35 |
| Covid-19 infection | 0.02 | 0.05 | 0.05 | 0.05 |
| Covid-19 infection relatives | 0.10 | 0.10 | 0.10 | 0.13 |
| Covid-19 death realtives | 0.03 | 0.05 | 0.05 | 0.04 |
| Violence | 0.00 | 0.02 | 0.02 | 0.00 |
| Stigmatization expectation | 0.14 | 0.23 | 0.25 | 0.11 |
| Stigmatization experience | 0.07 | 0.16 | 0.14 | 0.08 |
| Problem-focused coping | 0.13 | 0.29 | 0.24 | 0.55 |
| Escape-avoidant-focuses coping | 0.22 | 0.23 | 0.15 | 0.14 |
| Meaning-focused coping | 0.00 | 0.32 | 0.28 | 1.00 |
| Support-focused Coping | 0.22 | 0.29 | 0.27 | 0.37 |
| Extraversion | 0.54 | 0.57 | 0.57 | 0.59 |
| Conscientiousness | 0.64 | 0.62 | 0.64 | 0.66 |
| Agreeableness | 0.58 | 0.55 | 0.60 | 0.54 |
| Neuroticism | 0.62 | 0.59 | 0.55 | 0.52 |
| Openness | 0.60 | 0.63 | 0.61 | 0.69 |
| Needs: information | 0.38 | 0.38 | 0.30 | 0.36 |
| Needs: instrumental support | 0.15 | 0.34 | 0.22 | 0.15 |
| Needs: psychosocial support | 0.00 | 1.00 | 0.00 | 0.00 |
| Needs: child care | 0.14 | 0.13 | 0.11 | 0.12 |
| Needs: recommendations daily life | 0.22 | 0.34 | 0.29 | 0.22 |
| Needs: maintain well-being | 0.38 | 0.63 | 0.55 | 0.34 |
| Positive effects: none | 0.32 | 0.42 | 0.46 | 0.32 |
| Positive effects: social support | 0.43 | 0.38 | 0.35 | 0.45 |
| Positive effects: societal cohesion | 0.23 | 0.18 | 0.17 | 0.24 |
| Positive effects: political transparency | 0.19 | 0.16 | 0.14 | 0.23 |
| Positive effects: solidarity | 0.40 | 0.32 | 0.30 | 0.38 |
| Good family climate | 0.48 | 0.50 | 0.58 | 0.68 |
| Family climate change | 0.33 | 0.61 | 0.68 | 0.36 |
| Permanent relationship | 0.61 | 0.56 | 0.63 | 0.59 |
| Number household members | 0.39 | 0.40 | 0.43 | 0.37 |
| Children | 0.47 | 0.38 | 0.48 | 0.40 |
| Housing with balcony/terace/garden | 0.09 | 0.10 | 0.10 | 0.11 |
| Healthcare professional | 0.16 | 0.18 | 0.18 | 0.17 |
| COVID-19-related job constraints | 0.11 | 0.14 | 0.16 | 0.11 |
| Financial loss | 0.14 | 0.19 | 0.18 | 0.17 |
| Homeoffice | 0.29 | 0.28 | 0.24 | 0.28 |

**References**

1. Schmidt, S., Mühlan, H., & Power, M. (2005). The EUROHIS-QOL 8-item index: psychometric results of a cross-cultural field study. *European Journal of Public Health*, *16*(4), 420-428. <https://doi.org/10.1093/eurpub/cki155>.

2. Müller, W., Lüttinger, P., König, W., & Karle, W. (1989). Class and Education in Industrial Nations. *International Journal of Sociology*, *19*(3), 3-39.

3. Löwe, B., Spitzer, R. L., Zipfel, S., & Herzog, W. (2002). PHQ-D. Gesundheitsfragebogen für Patienten (PHQ-D). Manual Komplettversion und Kurzform. [PHQ-D. Patient Health Questionnaire (PHQ-D). Manual complete version and short form]. *2 Auflage*.

4. Richter, D., & Weinhardt, M. (2013). LS-S: Loneliness Scale-SOEP. In C. J. Kemper, M. Zenger, & E. Brähler (Eds.), Psychologische und sozialwissenschaftliche Kurzskalen: Standardisierte Erhebungsinstrumente für Wissenschaft und Praxis (1.st ed.). Berlin: Mwv Medizinisch Wissenschaftliche Verlagsges.

5. Bastien, C. H., Vallières, A., & Morin, C. M. (2001). Validation of the Insomnia Severity Index as an outcome measure for insomnia research. *Sleep Medicine*, *2*(4), 297-307. <https://doi.org/https://doi.org/10.1016/S1389-9457(00)00065-4>.

6. Cox, B., Oyen, H. V., Cambois, E., Jagger, C., Roy, S. l., Robine, J.-M., & Romieu, I. (2009). The reliability of the minimum European health module. *International Journal of Public Health*, *54*(2), 55-60. <https://doi.org/10.1007/s00038-009-7104-y>.

7. Schulze, B., Stuart, H., & Riedel-Heller, S. G. (2009). The German version of the Inventory of Stigmatizing Experiences (ISE) - a new tool for assessing the prevalence and impact of "felt stigma". *Psychiatrische Praxis*, *36*(8), e19-27. <https://doi.org/10.1055/s-0029-1223337>.

8. Carver, C. S. (1997). You want to measure coping but your protocol’ too long: Consider the brief cope. *International Journal of Behavioral Medicine*, *4*(1), 92. <https://doi.org/10.1207/s15327558ijbm0401_6>.

9. Rammstedt, B., Kemper, C. J., Klein, M. C., Beierlein, C., & Kovaleva, A. (2017). A Short Scale for Assessing the Big Five Dimensions of Personality: 10 Item Big Five Inventory (BFI-10). *2017*, *7*(2). <https://doi.org/10.12758/mda.2013.013>.
